# The causal relationship between psoriasis, psoriatic arthritis, and inflammatory bowel diseases

**DOI:** 10.1038/s41598-022-24872-5

**Published:** 2022-11-28

**Authors:** Yang Sun, Yue Li, Jiting Zhang

**Affiliations:** 1 Department of Orthopedics, The First Hospital of Jilin University, Changchun, Jilin China; 2 Department of Social Psychiatry, The Affiliated Brain Hospital of Guangzhou Medical University, Guangzhou, Guangdong China

**Keywords:** Inflammatory bowel disease, Skin diseases, Psoriasis

## Abstract

Psoriasis is more common in patients with inflammatory bowel disease (IBD) than in the general population. Similarly, patients with psoriasis or psoriatic arthritis (PsA) have a higher incidence of IBD. However, whether this association is causal remains unknown. Therefore, we used a two-sample bidirectional Mendelian randomization (MR) analysis to identify this relationship. According to MR analysis, psoriasis and PsA causally increased the odds of developing Crohn’s disease (OR = 1.350 (1.066–1.709) *P* = 0.013; OR = 1.319 (1.166–1.492) *P* < 0.001). In contrast, MR estimates gave little support to a possible causal effect of psoriasis, PsA, on ulcerative colitis (OR = 1.101 (0.905–1.340) *P* = 0.335; OR = 1.007 (0.941–1.078) *P* = 0.831). Similarly, the reverse analysis suggested the Crohn’s disease causally increased the odds of psoriasis and PsA (OR = 1.425 (1.174–1.731) *P* < 0.001; OR = 1.448 (1.156–1.182) *P* = 0.001), whereas there are no causal association between ulcerative colitis and psoriasis, PsA (OR = 1.192 (0.921–1.542) *P* = 0.182; OR = 1.166 (0.818–1.664) *P* = 0.396). In summary, our MR analysis strengthens the evidence for the bidirectional dual causality between psoriasis (including PsA) and Crohn’s disease.

## Introduction

As an inflammatory skin disorder, psoriasis is characterized by aberrant keratinocyte proliferation and immune cell infiltration into the epidermis^1^. Approximately 2.5% of Europeans, 0.05–3% of Africans, and 0.1–0.5% of Asians are affected^2–5^. Up to 30% of people with psoriasis eventually develop psoriatic arthritis (PsA), an inflammatory musculoskeletal condition^6^. PsA is one of the most severe comorbidities of psoriasis, which is characterized by joint pain, swelling and rigidity, and affects 0.4–1% of the UK population^7,8^. Psoriasis and PsA both have a significant genetic predisposition. Psoriasis has a heritability of 60–90%^9,10^, while PsA has a heritability of 80–100% based on twin and family research in European populations^11^. The disease prevalence is also on the rise^12^.

Inflammatory bowel disease (IBD), consisting of ulcerative colitis (UC) and Crohn’s disease (CD), is a chronic, recurrent immune-mediated disease of the gastrointestinal system^13^. The disease affects more than 2.5 million people in Europe, with rising prevalence in Asia and developing countries^14^. The association between IBD and psoriasis, PsA, has recently gained much attention. Specifically, several observational studies have investigated a strong relationship between psoriasis, PsA, and IBD, involving genetics, immunity, and gut dysbiosis^15–17^. Furthermore, a cohort study of US women found psoriasis with concomitant the psoriatic arthritis is associated with an increased risk of incident CD^16^.

In addition, according to a meta-analysis, psoriasis and IBD have been significantly linked in both directions, particularly in children and adolescents with IBD^18^. However, conclusions about causality cannot be drawn merely based on the presence of an association in an observational design, which was retrospective or cross-sectional in design with limited sample sizes and confounders. For example, patients with IBD treated with anti-TNF-α are susceptible to the side effects of paradoxical psoriasis, making medication a risk factor for psoriasis^19^. Therefore, it is difficult to determine whether PsA and IBD are causally related because this exaggerates the link between psoriasis and IBD.

Mendelian randomization (MR)^20^ has been proven to be a reliable method that can overcome observational studies’ limitations and assess causality. Traditional confounding factors are under control because the random allocation of alleles at conception ensures a balanced distribution of confounders across different genotypes. Furthermore, reverse causation is eliminated because a disease cannot alter a person’s genotype^20^.

In this study, we used the summary statistics from the public available genome-wide association studies (GWAS) data to conduct a bidirectional MR analysis to evaluate the potential causal relationship between psoriasis, PsA, and IBD.

## Methods

We used data from published studies or GWAS summaries that were openly available. Since no primary data were used in this study, ethical approval was not required. However, each study’s academic ethics review committees approved all of them, and each participant signed a written informed consent form.

### Selection of genetic variants and data sources

#### Genetic variants of psoriasis and PsA

Summary statistics for psoriasis and PsA were acquired from the MRC IEU OpenGWAS database (https://gwas.mrcieu.ac.uk/), which primarily consists of publicly available GWAS summary data and acts as an input source for many analytical methods, including Mendelian randomization^21,22^. All genetic variants reaching genome-wide significance (*P* < 5 × 10e−8) were selected as instruments for the MR analysis. The largest GWAS of psoriasis (N = 4510 cases, 212,242 controls) and PsA (N = 1637 cases, 212,242 controls) consisting of merely European individuals from the FinnGen biobank recovered some novel genetic variants. The corresponding linkage disequilibrium was tested to confirm any SNPs in linkage disequilibrium and whether the SNPs were independent by pruning SNPs within a 10,000 kb window with an r^2^ < 0.001 threshold. In addition, we examined the relationship between SNPs and potential confounders for the following traits: CD, UC, skin disorders, self-reported psoriasis, PsA, obesity, depression, disease duration, sex, race. SNPs with the abovementioned potential confounders were further eliminated.

#### Genetic variants of CD and UC

Summary statistics for CD and UC were acquired from the MRC IEU OpenGWAS database. We used a similar selection process to that described above to choose SNPs from the GWAS as the genetic instruments for CD and UC. As a result, several novel genetic variants were reclaimed from the largest GWAS of CD (N = 657 cases, 210,300 controls) and UC (N = 2251 cases, 210,300 controls) consisting of merely European individuals from the FinnGen biobank.

Finally, the F-statistic was calculated to assess the strength of the selected SNPs according to the following equation:

$$F=\frac{{\mathrm{R}}^{2}(N-1-K)}{\left(1-{\mathrm{R}}^{2}\right)K}$$

where R^2^ is the portion of exposure variance explained by the instrument variables (IVs), N is the sample size, and K is the number of IVs. F-statistic ≥ 10 suggests the non-existence of weak instrument bias^23^.

### Mendelian randomization estimates

We conducted eight separate two-sample MR analyses, evaluating the association results, to examine the genetically bidirectional causal effect between psoriasis, PsA, CD, and UC. The three main assumptions of the two-sample MR analysis are as follows^20,24^ (Supplementary Fig. S1):

Genetic variants are strongly associated with exposure.

The variants must affect the outcome only by exposure.

The variants must be unaffected by any confounding factors related to the exposure or outcome^25^.

Many robust methods have been proposed since not all genetic variants are valid IVs. The methods that contain inverse variance weighting (IVW), inverse variance weighted (fixed effects), maximum likelihood, penalized weighted median, weighted median, MR-Egger, weighted mode, and simple mode were based on different assumptions for MR analysis. However, the IVW method of each Wald estimate is the primary method for acquiring an MR assessment^26^.

The IVW method uses a meta-analysis approach to combine Wald estimates for each SNP to get the overall effect estimates^27^. An unbiased causal estimate can be obtained by IVW linear regression if the second assumption (no horizontal pleiotropy) is not violated or if the horizontal pleiotropy is balanced^22^. Fixed- and random-effects IVW approaches are available. If significant heterogeneity (*P* < 0.05) is observed, a random-effect IVW model is applied. Similar to the fixed-effects IVW approach, the maximum likelihood method assumes that there is no heterogeneity or horizontal pleiotropy. The effect of the SNP on the exposure is plotted against the effect of the SNP on the outcome using the MR-Egger method, and if pleiotropy is absent, the plotted points fall along a line that goes through the origin. Values of the intercept terms that are different from zero indicate pleiotropy.

Pleiotropy-corrected causal estimates can be obtained from the MR-Egger regression’s slope. This approach assumes that the horizontal pleiotropic effects are not correlated with the SNP-exposure effects (InSIDE assumption)^28^. MR-Egger regression places no limitations on the average value of the pleiotropic effects. It only requires the Instrument Strength Independent of Direct Effect (InSIDE) assumption to estimate the causal effect unbiasedly. Under the InSIDE assumption, the pleiotropic effects are independent of the variant–exposure associations^28^. The weighted median method computes the median and ranks the MR estimates produced by each instrument separately according to the inverse of their variances^29^. According to this method, only half of the SNPs must be valid instruments (i.e., exhibiting no horizontal pleiotropy, no association with confounders, and robust association with the exposure). This method improves precision compared to the MR-Egger regression method^29^.

In addition, a penalized weighted median was calculated where outlying variants are penalized. Even when most instrumental variables in the weighted model do not comply with the conditions for MR causal inference, the weighted model still performs well^30^. The simple mode is a model-based estimation method that provides robustness for pleiotropy, although it is not as powerful as IVW^31^.

### Sensitivity analysis

We used MR-Egger regression to assess potential pleiotropic effects that the SNPs used as IVs might have. The intercept term in MR-Egger regression can be a useful indication of whether directional horizontal pleiotropy is driving the results of an MR analysis^32^. MR-PRESSO is a method for detecting and correcting outliers in IVW linear regression. MR-PRESSO has three components: detection of horizontal pleiotropy (MR-PRESSO global test), correction for horizontal pleiotropy via outlier removal (MR-PRESSO outlier test), and testing of significant differences in the causal estimates before and after correction for outliers (MR-PRESSO distortion test). In brief, MR-PRESSO identifies horizontal pleiotropic outlier variants and provides an outlier-corrected estimate^33^. The heterogeneities was quantified by Cochran Q statistic; a *P* value of < 0.05 would be considered significant heterogeneity^25^. In addition, we conducted a “leave-one-out” sensitivity analysis, where the MR is left out individually to identify potentially significant SNP.

R version 4.0.5 with the two-sample MR and MR-PRESSO packages was used for all statistical analyses^22,33^. Statistical significance was defined as *P* value < 0.05.

## Results

### Selection of instrumental variables

The data on the LD-independent SNPs (after clumping) for exposures (psoriasis, PsA, CD, UC) are included in Supplementary Tables S1–S4. In the following cases, the listed SNPs will be removed: firstly, SNPs associated with outcomes and confounding factors will be excluded. Secondly, a specific SNP did not exist in the outcome GWAS, and a proxy in LD with the target SNP could not be retrieved from the outcome GWAS during extracting specific SNPs. Thirdly, it was impossible to reverse the impact of non-concordant alleles in ambiguous or palindromic SNPs with ambiguous strands. Finally, the F-statistics of IVs were all greater than 10, indicating little evidence of weak instrument bias (Supplementary Table S5).

### The causal effect of psoriasis and PsA on CD and UC

**Figure 1:**
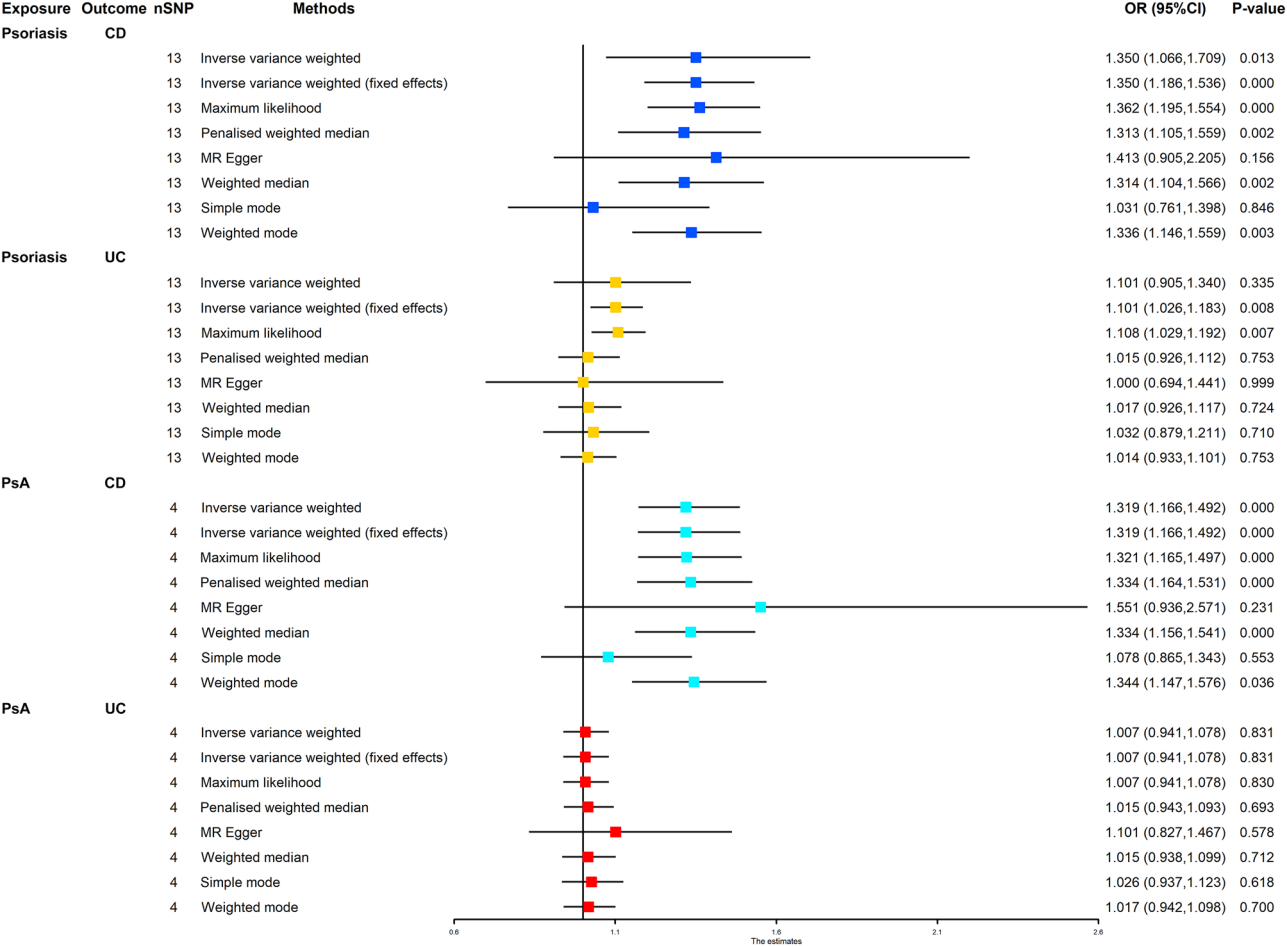
Forest plot for MR analyses of the causal effect of psoriasis and PsA on CD and UC. *CD* Crohn’s disease, *UC* ulcerative colitis, *PsA* psoriatic arthritis, *nSNP* number of single nucleotide polymorphism.

**Figure 2:**
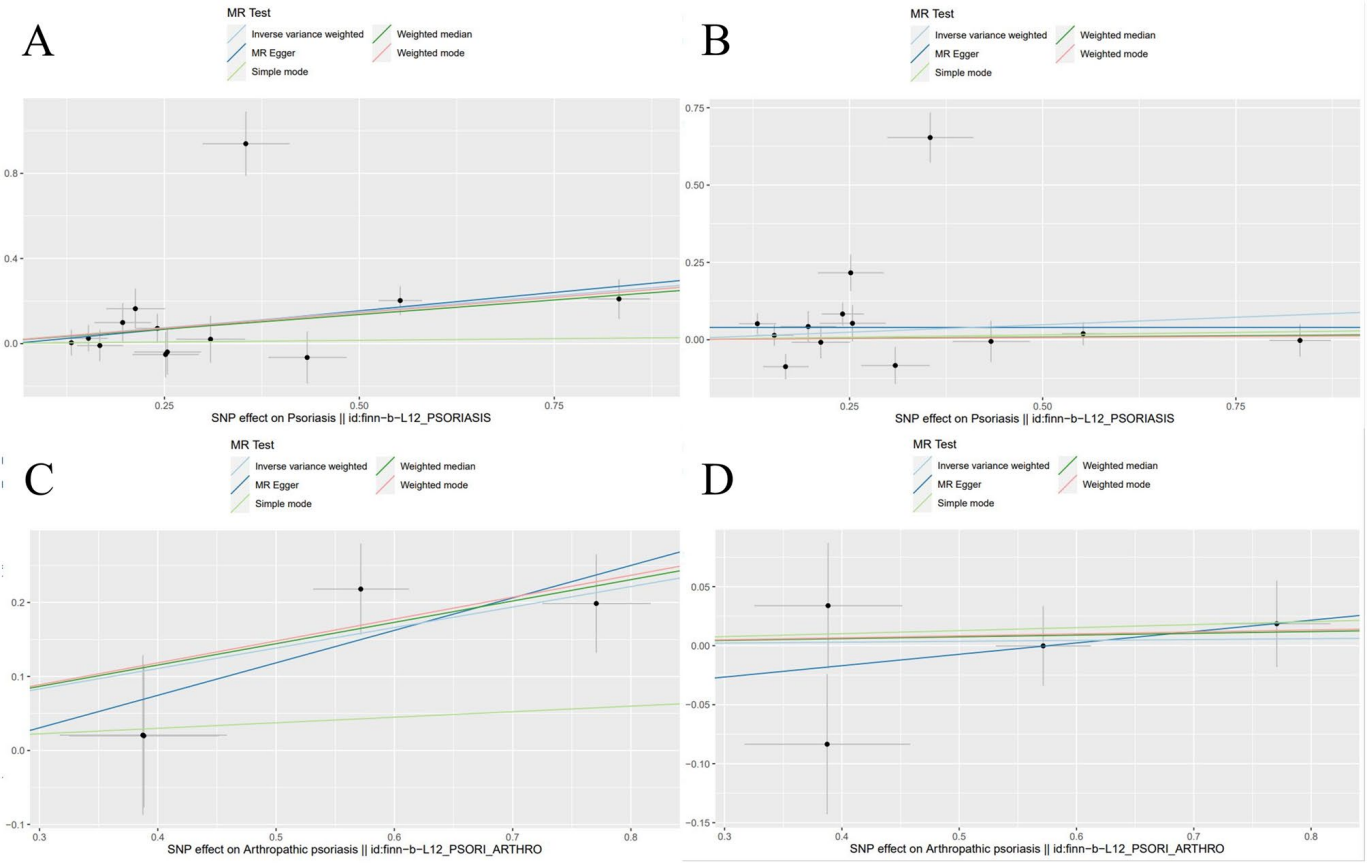
Scatter plots for MR analyses of the causal effect of psoriasis and PsA on CD and UC. (**A**) Scatter plots for MR analyses of the causal effect of psoriasis on CD; (**B**) scatter plots for MR analyses of the causal effect of psoriasis on UC; (**C**) scatter plots for MR analyses of the causal effect of PsA on CD; (**D**) scatter plots for MR analyses of the causal effect of PsA on UC. *MR* Mendelian randomization, *CD* Crohn’s disease, *UC* ulcerative colitis, *PsA* psoriatic arthritis, *SNP* single nucleotide polymorphism.

Figure 1 shows the results of estimating the causal effect of psoriasis and PsA on CD and UC. It demonstrated that psoriasis was associated with a 35% increased risk of CD (IVW: OR 1.350 (1.066–1.709), *P* = 0.013). The association was consistent in the maximum likelihood, penalized weighted median, weighted median, and weighted mode methods. However, no causal effect of psoriasis on UC was found (IVW: OR 1.101 (0.905–1.340), *P* = 0.335). Since the MR assessment of PsA on CD and UC did not show any heterogeneity (Table 1), IVW with a fixed effect was identified as the main MR analysis. Primary MR analysis by the IVW (fixed effects) method showed that PsA was associated with the 31.9% increased risk of CD (IVW (fixed effects): OR 1.319 (1.166–1.492), *P* < 0.001). However, there was no causal genetic association between PsA and UC (IVW (fixed effects): OR 1.007 (0.941–1.078), *P* = 0.831). The estimated effect sizes of the SNPs on both exposure and result were displayed using scatter plots (Fig. 2). Supplementary Figs. S2 and S3 displays the “leave-one-out analysis” plots and funnel plots.

**Table 1:** Heterogeneity test and horizontal pleiotropy test.

Exposure	Outcome	Heterogeneity test (IVW)		Heterogeneity test (MR-Egger)		Horizontal pleiotropy test	
		Q	*P* value	Q	*P* value	Q	*P* value
Psoriasis	CD	40.023	0.000	39.815	0.000	− 0.017	0.815
Psoriasis	UC	90.941	0.000	87.875	0.000	0.040	0.548
Psoriasis arthritis	CD	2.501	0.475	2.067	0.356	− 0.101	0.583
Psoriasis arthritis	UC	2.264	0.453	2.192	0.334	− 0.055	0.594
CD	Psoriasis	48.129	0.000	33.543	0.000	0.222	0.336
CD	Psoriasis arthritis	24.962	0.000	23.664	0.000	0.106	0.712
UC	Psoriasis	46.033	0.000	26.831	0.000	− 0.134	0.166
UC	Psoriasis arthritis	33.782	0.000	26.096	0.000	− 0.136	0.339

*IVW* inverse variance weighting, *MR* Mendelian randomization, *CD* Crohn's disease, *UC* ulcerative colitis.

### Estimates of the causal effect of CD and UC on psoriasis and PsA

**Figure 3:**
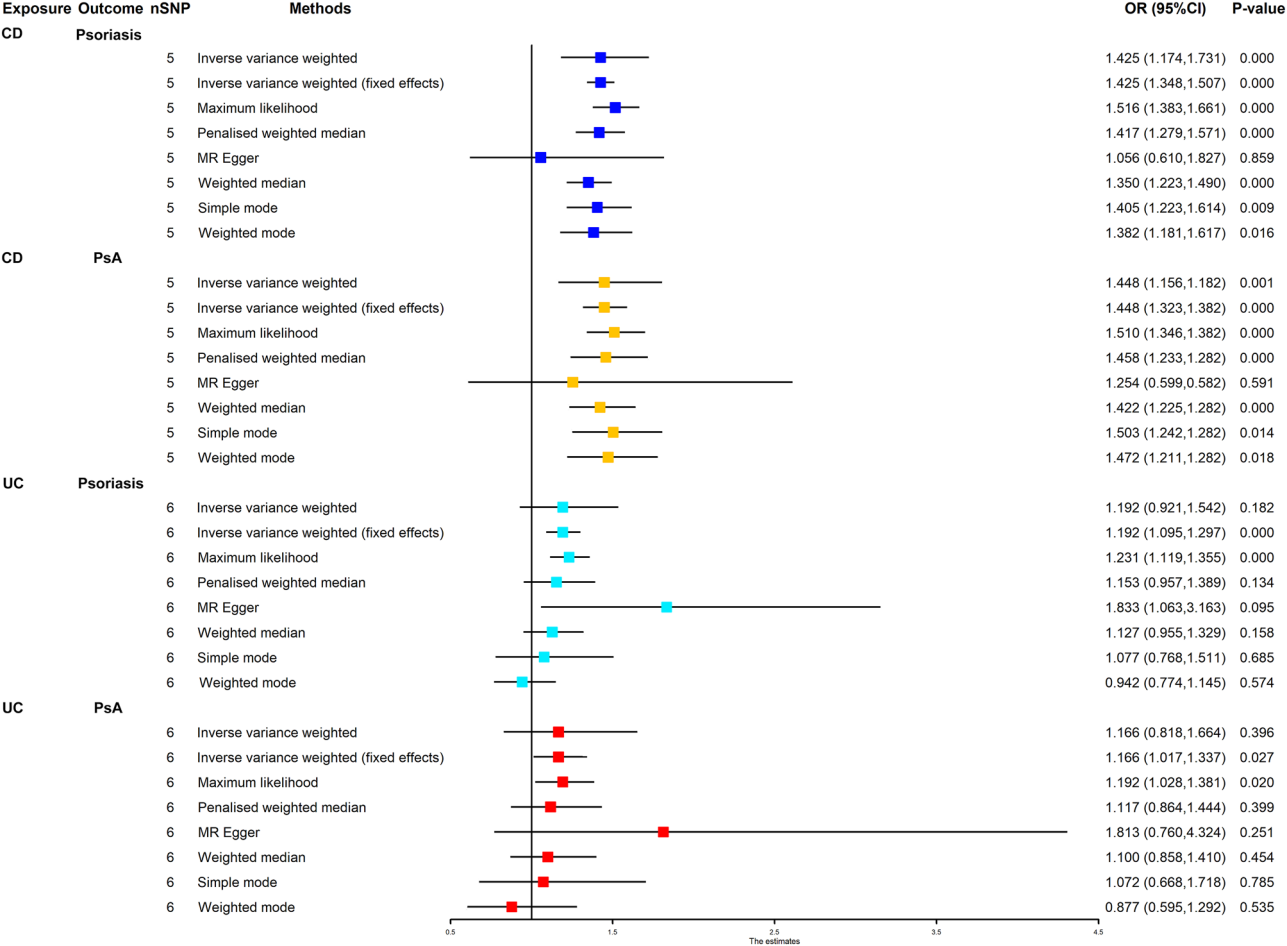
Forest plot for MR analyses of the causal effect of CD and UC on psoriasis and PsA. *CD* Crohn’s disease, *UC* ulcerative colitis, *PsA* psoriatic arthritis, *nSNP* number of single nucleotide polymorphism.

**Figure 4:**
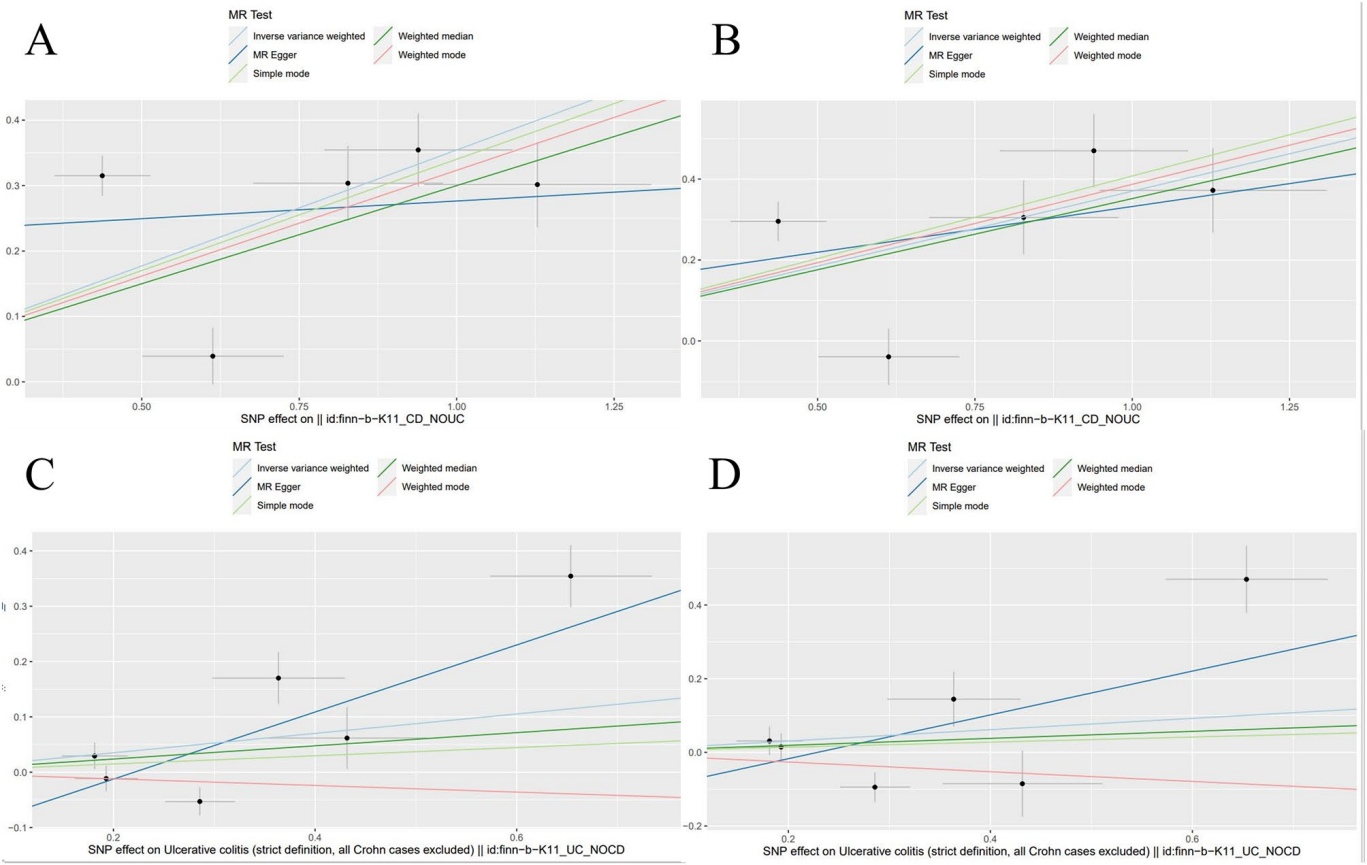
Scatter plots for MR analyses of the causal effect of CD and UC on psoriasis and PsA. (**A**) scatter plots for MR analyses of the causal effect of CD on psoriasis; (**B**) scatter plots for MR analyses of the causal effect of CD on PsA; (**C**) scatter plots for MR analyses of the causal effect of UC on psoriasis; (**D**) scatter plots for MR analyses of the causal effect of UC on PsA. *MR* Mendelian randomization, *CD* Crohn’s disease, *UC* ulcerative colitis, *PsA* psoriatic arthritis, *SNP* single nucleotide polymorphism.

Figure 3 displays MR estimates from eight different techniques for determining how CD and UC cause psoriasis and PsA. We found that genetically predicted CD was positively associated with the 42.5% increased risk of psoriasis (IVW: OR 1.425 (1.174–1.731), *P* < 0.001). The association was consistent in maximum likelihood, penalized weighted median, weighted median, simple mode, and weighted mode methods. In addition, the findings showed that CD was linked to a 44.8% higher risk of PsA (IVW: OR 1.448 (1.156–1.812), *P* = 0.001). The association was consistent in the maximum likelihood, penalized weighted median, weighted median, simple mode, and weighted mode methods. However, no causal effect of UC on psoriasis was found (IVW: OR 1.192 (0.921–1.542), *P* = 0.182). Similarly, there was no causal genetic association between UC and PsA (IVW: OR 1.166 (0.818–1.664), *P* = 0.396). Figure 4 and Supplementary Figs. S4 and S5 show the scatter plots, funnel plots, and “leave-one-out analysis” plots.

### Sensitivity analysis

In MR analyses, the heterogeneity test indicated the existence of heterogeneity except for assessing PsA on CD and UC **(**Table 1). In addition, none of the MR analyses’ MR-Egger intercept evidence for horizontal pleiotropy (Table 1). Notably, the raw estimates from MR-PRESSO showed no association between psoriasis and CD. However, after excluding two SNPs, the outlier-corrected estimates yielded the opposite conclusion, identical to the result of the IVW, maximum likelihood, penalized weighted median, weighted median, and weighted mode method. Results from IVW or IVW (fixed) methods were consistent with the raw and outlier-corrected estimates from MR-PRESSO in the remaining analysis, demonstrating the stability of the results (Table 2).

**Table 2:** MR-PRESSO estimates between psoriasis, psoriatic arthritis and CD, UC.

Exposure	Outcome	Raw estimates				Outlier corrected estimates			
		N	OR	95%CI	*P* value	N	OR	95%CI	*P* value
Psoriasis	CD	13	1.188	(0.972, 1.452)	0.115	11	1.249	(1.139, 1.369)	0.000
Psoriasis	UC	13	1.054	(0.913, 1.217)	0.482	11	1.012	(0.946, 1.082)	0.738
Psoriasis arthritis	CD	4	1.319	(1.179, 1.476)	0.017	NA	NA	NA	NA
Psoriasis arthritis	UC	4	1.007	(0.946, 1.073)	0.834	NA	NA	NA	NA
CD	Psoriasis	5	1.425	(1.174, 1.731)	0.023	3	1.395	(1.299, 1.498)	0.012
CD	Psoriasis arthritis	5	1.448	(1.156, 1.812)	0.032	3	1.492	(1.344, 1.656)	0.017
UC	Psoriasis	6	1.192	(0.921, 1.542)	0.240	4	1.182	(0.951, 1.469)	0.230
UC	Psoriasis arthritis	6	1.166	(0.818, 1.664)	0.435	4	1.119	(0.884, 1.416)	0.420

*MR* Mendelian randomization, *CD* Crohn's disease, *UC* ulcerative colitis, *N* number, *OR* odds ratio, *CI* confidence interval.

## Discussion

A bidirectional causal relationship was found between psoriasis, PsA, and CD, but not between psoriasis, PsA, and UC. Results showed that psoriasis and PsA were associated with 35% and 31.9% increased CD risk, respectively. Similarly, genetically predicted CD was positively associated with the increased risks of 42.5% and 44.8% of psoriasis and PsA, respectively. However, there were no correlations between UC and psoriasis or PsA.

According to our findings, there is a bidirectional causal relationship between psoriasis, PsA, and CD. These findings align with previous studies^16,34,35^ and could be explained by the fact that these conditions have similar pathogenesis. Firstly, they have common genetic risk loci. More than 4500 cases and 10,000 controls were investigated in GWAS, where 7 non-HLA susceptibility loci shared by CD and psoriasis (9p24 near JAK2, 10q22 at ZMIZ1, 11q13 near PRDX5, 16p13 near SOCS1, 19p13 near FUT2, 17q21 at STAT3, 22q11 at YDJC) were found. Four previously identified common risk loci (IL23R, IL12B, REL, and TYK2) were affirmed^36^. Chromosomal locus 6p21, the most widely researched genetic region, encompasses the major histocompatibility complex (MHC)-related genes^37^, which corresponds to PSORS1 in psoriasis and IBD3 in IBD^38^. Secondly, patients with CD and psoriasis frequently have gut dysbiosis^39^. A gut-skin-joint axis has been proposed by researchers to shed light on the relationships between variations in gut microbiota, increased bowel permeability, and disturbed immune balance, which may cause inflammation of the skin and joints^40^. Gut microbiota will affect epidermal divergence signaling pathways to change skin homeostasis^41^. In addition, some bacteria, like parabacteroides and coprobacillus, are less common due to CD and psoriasis^42^. Thirdly, immunological mechanisms that link psoriasis and CD may be dysbiosis, which dysbiosis possibly acting as a common pathogenic pathway that causes an augmented Th17-driven immune response in genetically susceptible hosts^43^. Furthermore, IL-23 could promote the proliferation and survival of Th17 cells while also inducing the release of corresponding cytokines, thus serving as a crucial cytokine regulator in autoimmune disorders^44,45^. Patients with PsA are more likely to have an autoimmune disorder than those with only cutaneous disease^46^, which could be attributed to the fact that patients with PsA have higher levels of systemic inflammation than those with psoriasis^46,47^. The negative results associated with UC were consistent with the findings of several previous studies^16,34,35^. However, various studies have demonstrated associations between psoriasis, PsA, and UC, while some report a higher risk of CD than UC^17,48,49^. Despite the clinical signs, genetic risk loci, and immune pathways shared by CD and UC, they have distinctive properties that may illustrate the discrepancy in their link with psoriasis and PsA.

Since our results seem to be interesting in the literature there are a few studies which present similar results. Yajia et al. have presented another MR analysis which confirms the bidirectional relationship between psoriasis, psoriatic arthritis, and CD^50^. However, findings of Dennis' study only support a unidirectional causal effect between CD and psoriasis as well as psoriatic arthritis^51^. There are some discrepancies between these reported estimates and our results. We believe that the difference may be caused by the different data source themselves. Notably these studies (including our results) all denied a causal association between UC and psoriasis (including PsA).

Since many studies were cross-sectional or retrospective, it was difficult to determine the timing of the diagnosis of psoriasis and PsA with IBD. Although subsequent cohort studies have emerged, they can still not circumvent the effects of confounding factors. Therefore, we applied the Mendelian randomization study to circumvent these shortcomings. The robustness and reliability of our results were also improved by using multiple statistical approaches based on different assumptions for two-sample MR analysis. In addition, the summary GWAS data we extracted for psoriasis, PsA, and IBD were all from subjects of European lineage, reducing potential bias. More importantly, we provide a basis for scientific exploration between psoriasis (including PsA) and IBD.

Our study also has some limitations. Firstly, since each method we applied in the analyses has its advantages and disadvantages, there is a risk of obtaining inconsistent results. Fortunately, the results of all methods we have applied are consistent in direction, with only a few statistically insignificant. Secondly, as with all MR studies, we could not address unobserved pleiotropy. Thirdly, as our data are drawn from publicly published databases, the number of IVs screened is still limited despite our selection of the largest publicly published GWAS. This requires us to track relevant databases and update our data in time. Fourthly, the study population only included individuals of European ancestry. More studies should be conducted to verify the applicability of these results to other ethnicities.

## Conclusion

Our MR analysis strengthens the evidence for the bidirectional causal relationship between psoriasis (including PsA) and Crohn’s disease. Furthermore, the results suggest a compelling rationale for the clinician to detect the earlier potential development of these diseases.

## Supplementary Information

Supplementary Information 1.

Supplementary Information 2.

## Data Availability

All data used during the study were provided by a third party (GWAS summary data: https://gwas.mrcieu.ac.uk/). Direct requests for these materials may be made to the provider as indicated in the Acknowledgments.

## Abbreviations

PsA: Psoriatic arthritis
IBD: Inflammatory bowel disease
UC: Ulcerative colitis
CD: Crohn's disease
MR: Mendelian randomization
GWAS: Genome-wide association studies
IV: Instrument variable
IVW: Inverse variance weighting
SNP: Single nucleotide polymorphism
