# Supplementary material for: The causal relationship between psoriasis, psoriatic arthritis, and inflammatory bowel diseases
Source: Sci Rep. 2022 Nov 28;12:20526. doi: 10.1038/s41598-022-24872-5 (PMC9705442; doi:10.1038/s41598-022-24872-5)
Supplement: Supplementary file 1 — Supplementary Information 1. [file 41598_2022_24872_MOESM1_ESM.docx]

**The causal relationship between psoriasis, psoriatic arthritis and inflammatory bowel diseases**

Supplementary Materials

**Table S1.** **SNPs (after clumping) for psoriasis.**

**Table S2. SNPs (after clumping) for psoriatic arthritis.**

**Table S3** **SNPs (after clumping) for crohn's disease.**

**Table S4. SNPs (after clumping) for ulcerative colitis.**

**Table S5. The F-statistics of instrument variables.**

**Figure S1. Schematic representation of Mendelian randomization analysis.**

**Figure S2. Funnel plots for the causal effect of psoriasis, PsA on CD, UC.**

**Figure S3. “leave-one-out analysis” plots** **for the causal effect of psoriasis, PsA on CD, UC.**

**Figure S4. Funnel plots for the causal effect of CD, UC on psoriasis, PsA.**

**Figure S5. “leave-one-out analysis” plots for the causal effect of CD, UC on psoriasis, PsA.**

**Table S1. SNPs (after clumping) for psoriasis.**

| SNP | effect_allele | other_allele | beta | se | eaf | pval | gene |
| --- | --- | --- | --- | --- | --- | --- | --- |
| rs12713428 | C | A | 0.1694 | 0.0261 | 0.2487 | 8.11E-11 | REL |
| rs17728338 | A | G | 0.3092 | 0.0439 | 0.07331 | 1.76E-12 | ANXA6 |
| rs12188300 | T | A | 0.4331 | 0.0495 | 0.05898 | 2.24E-18 | AC008697.1 |
| rs674451 | C | T | 0.1307 | 0.0235 | 0.343 | 2.82E-08 | AL356739.1 |
| rs13210419 | A | G | 1.1157 | 0.0511 | 0.05858 | 1.1E-105 | HLA-B |
| rs28752856 | G | C | 0.833 | 0.0393 | 0.1106 | 5.9E-100 | - |
| rs4947309 | T | A | 0.5524 | 0.0274 | 0.2334 | 3.86E-90 | - |
| rs4713605 | A | T | 0.1526 | 0.0241 | 0.3321 | 2.35E-10 | TBC1D22B |
| rs9481169 | T | G | 0.2515 | 0.0422 | 0.07817 | 2.47E-09 | TRAF3IP2 |
| rs1611309 | T | C | 0.241 | 0.0267 | 0.6266 | 1.6E-19 | HLA-U |
| rs60600003 | G | T | 0.2128 | 0.0372 | 0.1017 | 1.03E-08 | ELMO1 |
| rs181316459 | C | G | 0.3544 | 0.0553 | 0.04736 | 1.5E-10 | FBXL18 |
| rs10829130 | A | G | 0.1965 | 0.0359 | 0.11 | 4.24E-08 | snoU13 |
| rs138009430 | A | C | 0.2538 | 0.0423 | 0.07849 | 1.94E-09 | FLJ21408 |
| rs2021511 | T | C | -0.1387 | 0.0254 | 0.2664 | 4.75E-08 | RMI2 |
| rs28998802 | A | G | 0.1672 | 0.0289 | 0.1851 | 7.41E-09 | NOS2 |

**Table S2. SNPs (after clumping) for psoriatic arthritis**

| SNP | effect_allele | other_allele | beta | se | eaf | pval | gene |
| --- | --- | --- | --- | --- | --- | --- | --- |
| rs17728338 | A | G | 0.3874 | 0.0704 | 0.07309 | 3.83E-08 | ANXA6 |
| rs28752869 | T | A | 0.7707 | 0.0454 | 0.2322 | 1.64E-64 | HLA-B |
| rs115174302 | T | C | 0.3882 | 0.0628 | 0.09402 | 6.34E-10 | ZFP57 |
| rs9281366 | A | T | 0.5716 | 0.0402 | 0.3059 | 7.79E-46 | HLA-B |
| rs2523560 | T | C | -0.3091 | 0.0391 | 0.5078 | 2.9E-15 | DHFRP2 |

**Table S3 SNPs (after clumping) for crohn's disease.**

| SNP | effect_allele | other_allele | beta | se | eaf | pval | gene |
| --- | --- | --- | --- | --- | --- | --- | --- |
| rs9380317 | T | C | 0.4374 | 0.0755 | 0.1974 | 6.92E-09 | - |
| rs139805598 | T | C | 1.1282 | 0.1799 | 0.03128 | 3.57E-10 | SLC29A4 |
| rs34022406 | C | T | 0.8274 | 0.1502 | 0.04245 | 3.63E-08 | TNRC18 |
| rs181316459 | C | G | 0.939 | 0.1489 | 0.0468 | 2.87E-10 | FBXL18 |
| rs112456327 | T | C | 0.6129 | 0.1115 | 0.07661 | 3.86E-08 | BRD7 |

**Table S4. SNPs (after clumping) for ulcerative colitis.**

| SNP | effect_allele | other_allele | se | beta | eaf | pval | gene |
| --- | --- | --- | --- | --- | --- | --- | --- |
| rs7553638 | G | A | 0.0308 | -0.1928 | 0.4913 | 3.7E-10 | OTUD3 |
| rs9275160 | A | G | 0.0342 | -0.2857 | 0.2966 | 7.32E-17 | TBC1D22B |
| rs75144213 | T | G | 0.0786 | 0.4317 | 0.04231 | 3.98E-08 | CYP3A54P |
| rs10807943 | C | T | 0.0656 | -0.3637 | 0.9371 | 2.92E-08 | SLC29A4 |
| rs181316459 | C | G | 0.0797 | 0.6536 | 0.04697 | 2.34E-16 | FBXL18 |
| rs11078921 | A | C | 0.0324 | 0.1813 | 0.3448 | 2.21E-08 | IKZF3 |

**Table S5. The F-statistics of instrument variables.**

| Exposure | Outcome | NO.SNP | F-statistics |
| --- | --- | --- | --- |
| Psoriasis | CD | 15 | 39.353 |
| Psoriasis | UC | 15 | 39.353 |
| Psoriatic arthritis | CD | 4 | 49.851 |
| Psoriatic arthritis | UC | 4 | 49.851 |
| CD | Psoriasis | 5 | 4.662 |
| CD | Psoriatic arthritis | 5 | 4.662 |
| UC | Psoriasis | 6 | 12.494 |
| UC | Psoriatic arthritis | 6 | 12.494 |


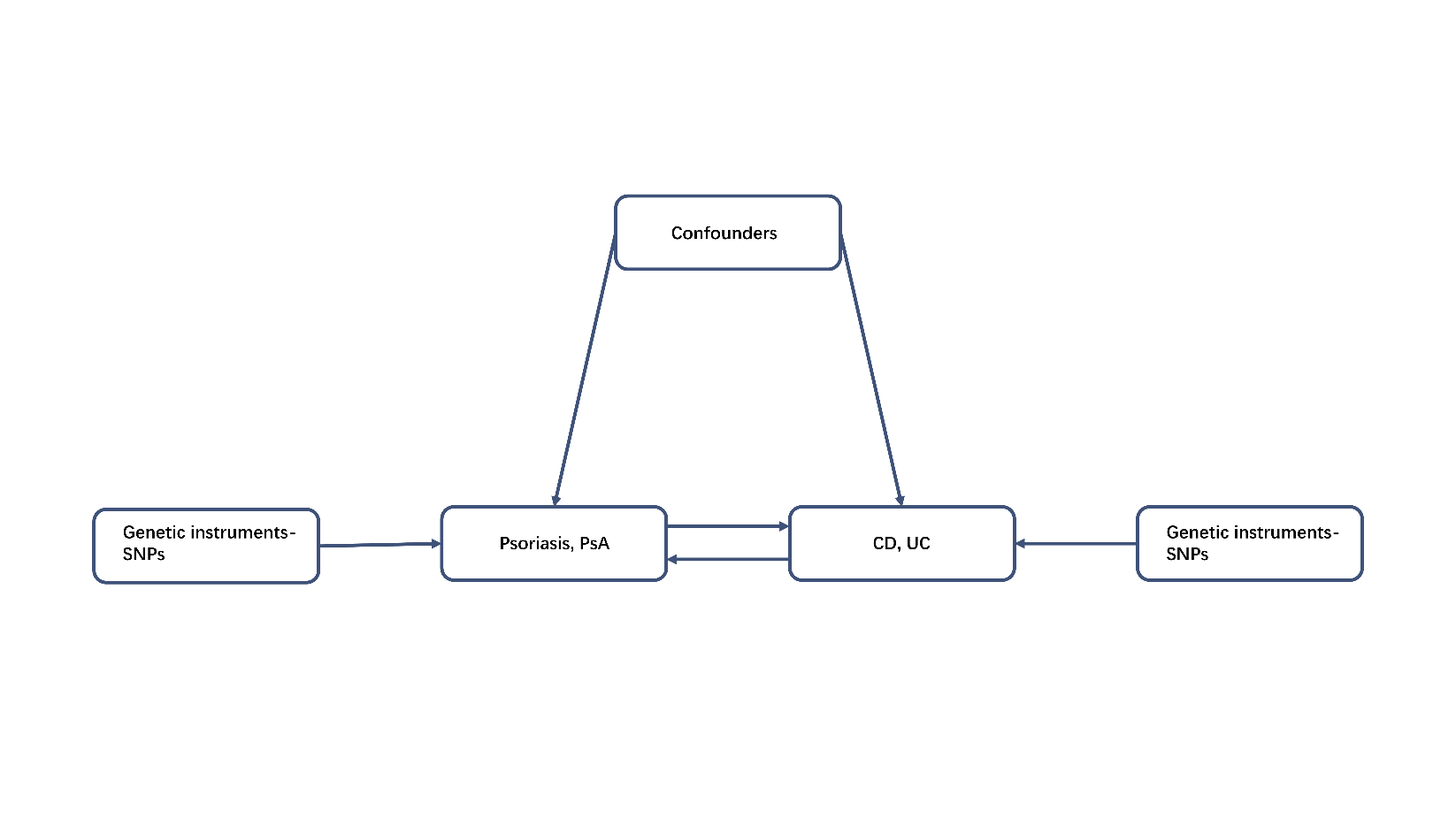


**Figure S1.** Schematic representation of Mendelian randomization analysis. PsA, psoriatic

arthritis; CD, crohn's disease; UC, ulcerative colitis; SNP, single nucleotide polymorphism.


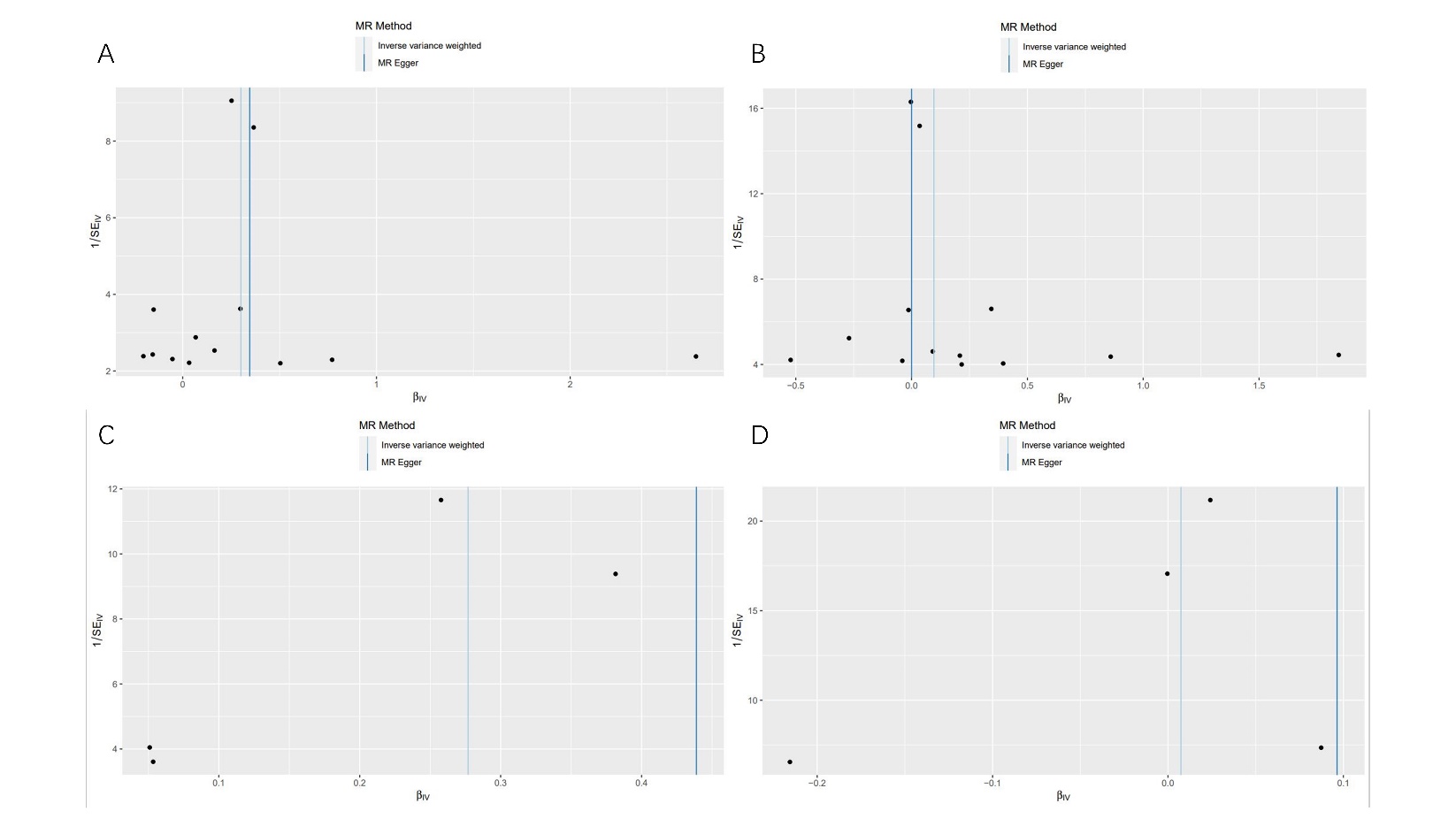


**Figure S2.** Funnel plots for the causal effect of psoriasis, PsA on CD, UC. A. psoriasis on CD; B. psoriasis on UC; C. PsA on CD; D. PsA on UC.


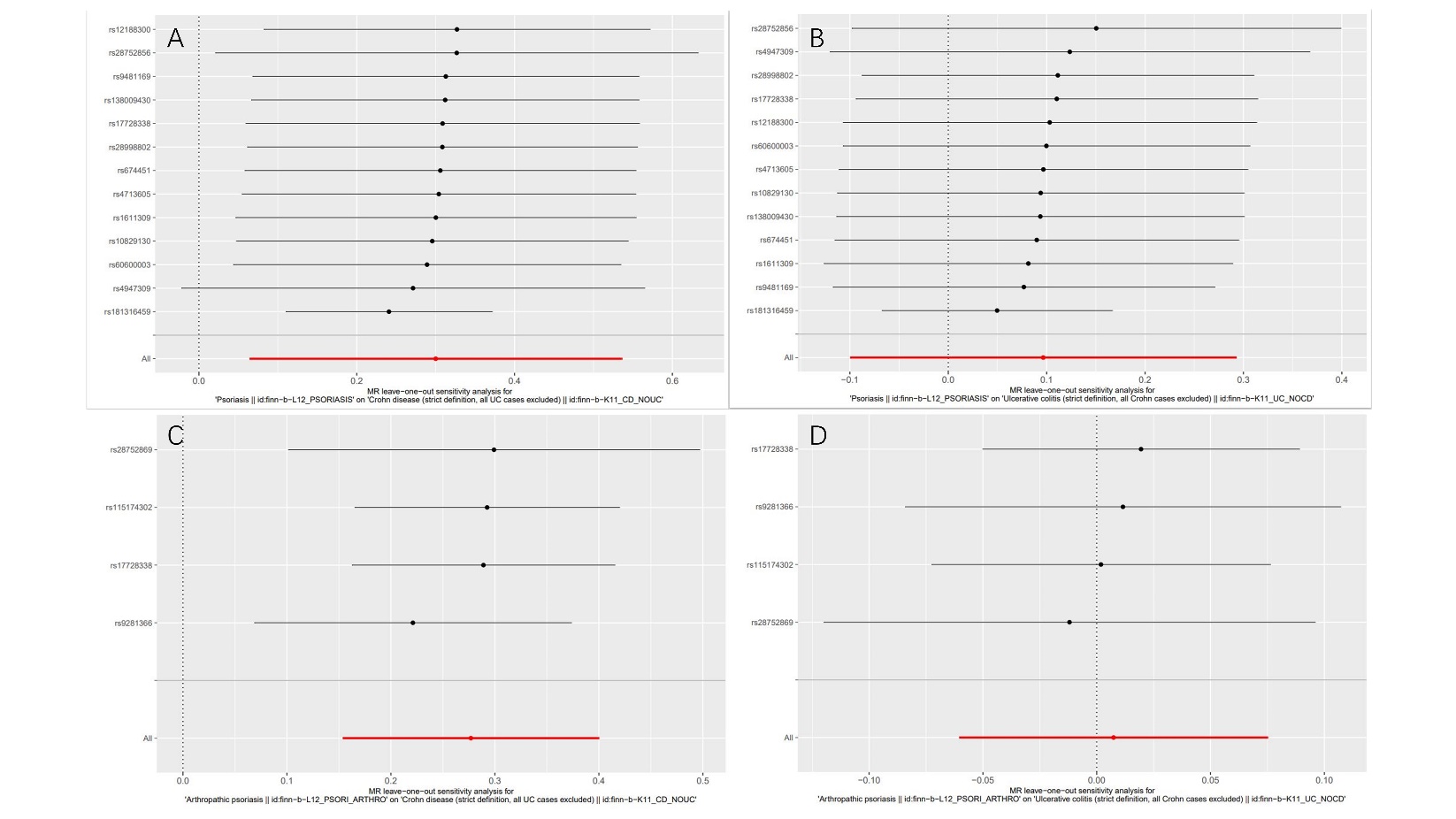


**Figure S3.** “leave-one-out analysis” plots for the causal effect of psoriasis, PsA on CD, UC. A. psoriasis on CD; B. psoriasis on UC; C. PsA on CD; D. PsA on UC.


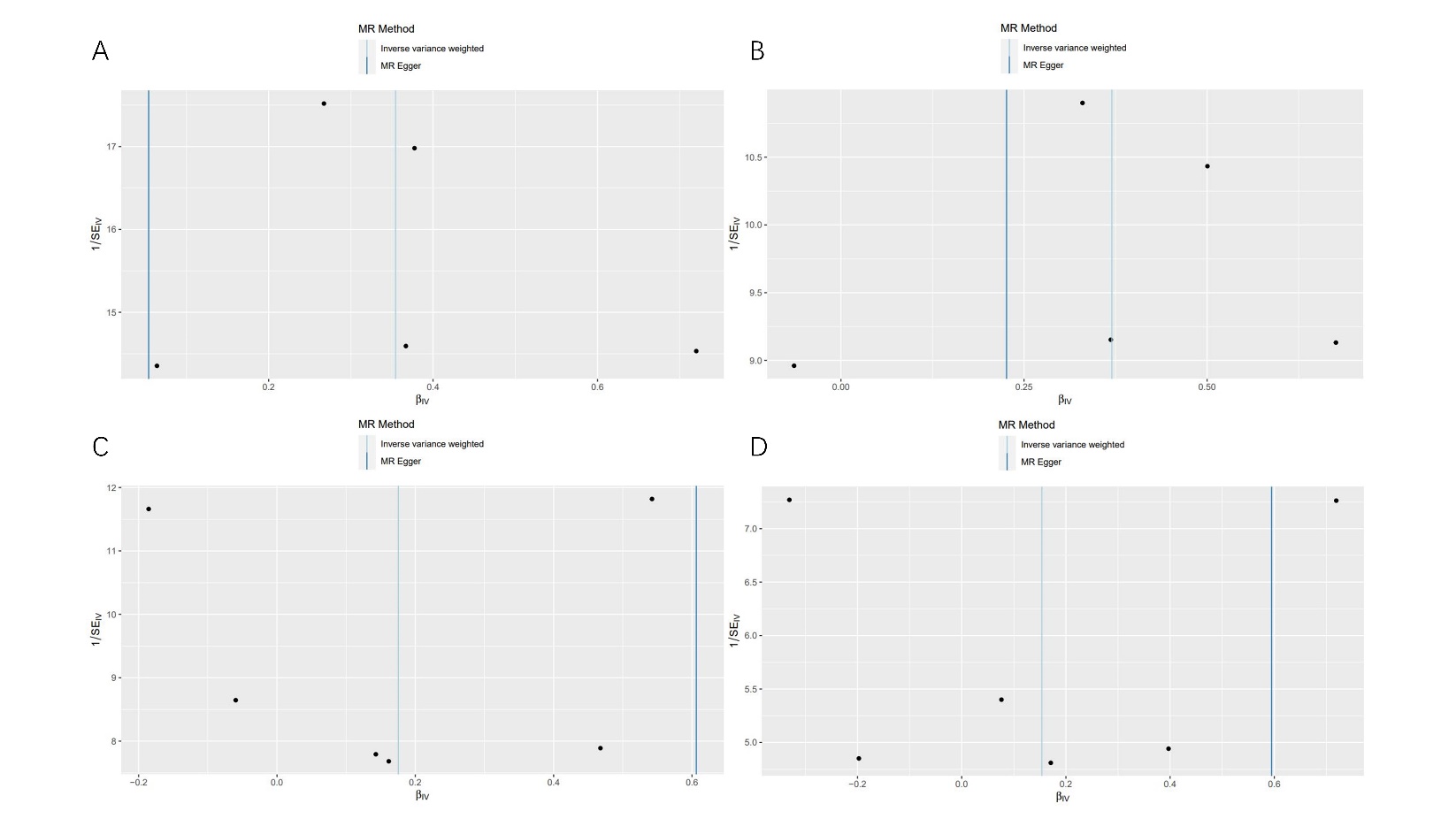


**Figure S4.** Funnel plots for the causal effect of CD, UC on psoriasis, PsA. A. CD on psoriasis; B. CD on PsA; C. UC on psoriasis; D. UC on PsA.


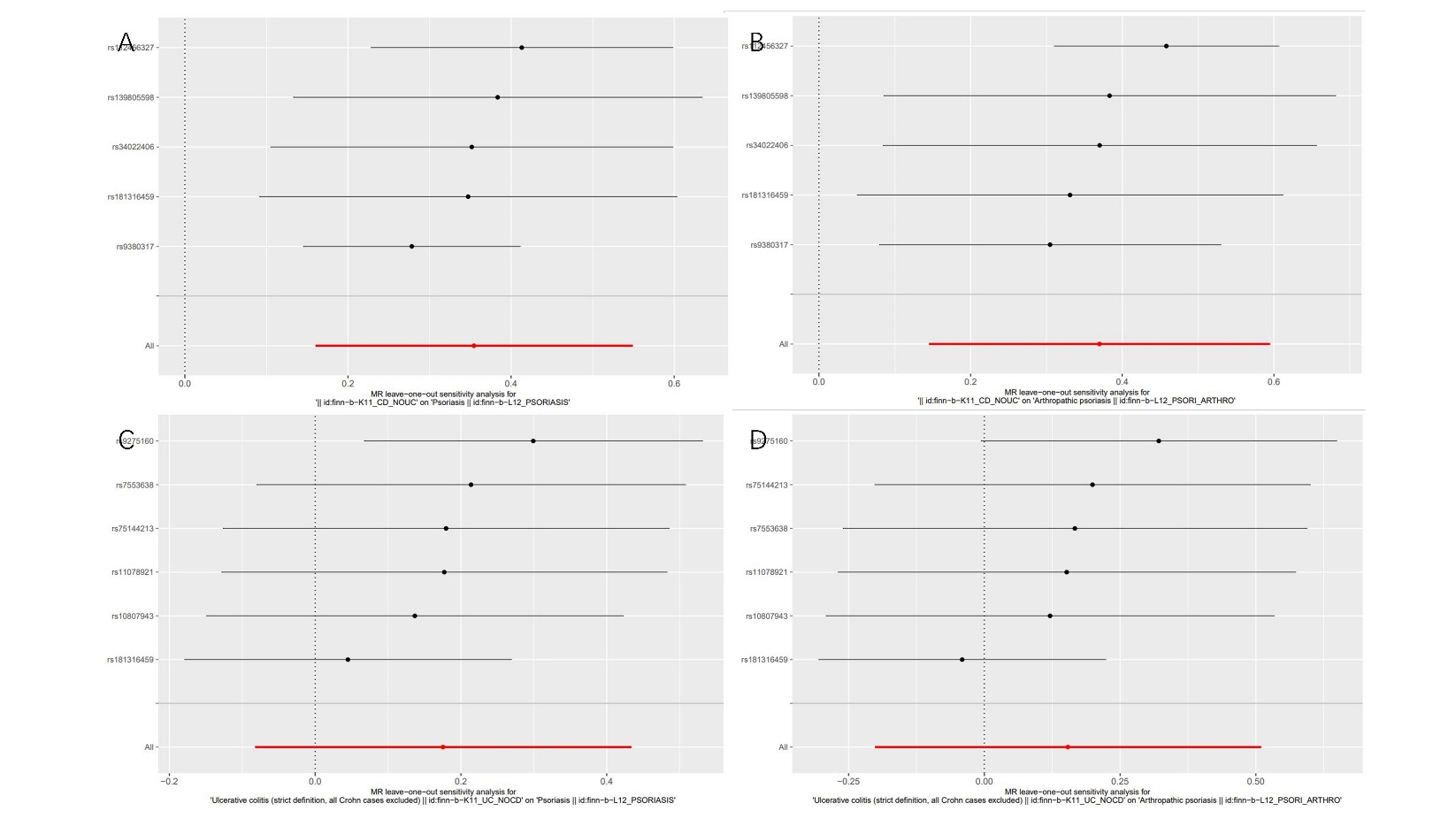


**Figure S5.** “leave-one-out analysis” plots for the causal effect of CD, UC on psoriasis, PsA. A. CD on psoriasis; B. CD on PsA; C. UC on psoriasis; D. UC on PsA.
